# The Administration of the Salop Infirmary

**Published:** 1903-04-04

**Authors:** 


### THE ADMINISTRATION OF THE SALOP INFIRMARY.

### BUSINESS, CHARITY, OR NEITHER?

The finances of the Salop Infirmary have for some years past given a good deal of anxiety to its managers. On June 30th last its accounts showed a deficit of over £2,112, even though the funds had been helped by a legacy of £2,000 from the late Sir Henry Edwardes. This deficit was growing annually, and in the circumstances the trustees resolved to seek expert advice as to how they could either diminish the expenses or increase the income. To this end the Bishop of Shrewsbury wrote to Sir Henry Burdett in the name of a special committee of trustees and subscribers, asking him to investigate the administrative position of the hospital and report thereon.

The report which was laid before the committee on March 14th dealt exclusively with the patients, terms of admission, and funds of the hospital, although Sir Henry, in the remarks he made warned those present that the time could not be far off when they would have to consider the question of rebuilding the infirmary —a sufficiently alarming prospect to an institution which cannot pay its way. With regard to the hospital expenditure Sir Henry had no fault to find. The housekeeping, indeed, is distinctly economical. The board for each in-patient costs 5s. a week, and that of the officials 8s. a week. This is nom ore than what it cost seven years ago, although the prices of many commodities have increased in the interval. The average cost of provisions per occupied bed—£21—differs but little from that of other provincial hospitals of the same size. It might seem as if in Chester Infirmary, where the cost of food is only £17 5s. per occupied bed, there was a greater economy, but in that institution the average, per occupied bed, for salaries, is nearly £5 higher than at the Salop Infirmary. This apparent wide divergence on these two points means, however, only that the two hospitals keep their accounts in a different way, and that the board of the officials is set down to housekeeping in the one institution, and added to the sum of salaries in the other. The Salop managers have resolved, on Sir Henry Burdett's advice, to adopt the uniform system of accounts, and if all hospitals would agree to follow this system it would be much easier than it is now for the managers of one place to glean hints as to economy in management from those of another.

But it is not the management of the Salop Infirmary that is at fault; it is its income, and the way in which the income is raised. It is a ticket hospital. Hitherto every annual subscriber of a guinea has had the right to one in-patient or four out-patient letters. The average cost of an in-patient is £4 12s. Therefore the subscriber of a guinea, if he avails himself of his privilege of recommendation, gets from the hospital about four and a-half times as much as he gives. That he does not receive this benefit himself is a minor matter. He gets the credit of it from the person who benefits. The result of this system is shown by the fact that while the guinea subscribers contributed £964 19s. to the fund of the infirmary, the patients whom they recommended cost £4,227. If each subscriber gets four and a-half times what he pays for, the result to the hospital can easily be guessed. On the other hand, it is estimated that an out-patient costs the hospital where he is treated about 1s. 6d. Yet a guinea subscription earned only four out-patient letters. Thus the subscriber contributes 5s. 3d. for every out-patient he recommends, or more than three times the amount of the cost of treatment. We need not assume that each subscriber determines to get as much as he can out of the hospital. There is probably little thought on the matter on the part of either the donors or the managers. But a ticket hospital is more or less managed on a business basis, and the Salop Infirmary basis has hitherto been one of ill-managed business. Some people get too much for their money; others get too little. It is, as Sir Henry said, neither a business nor a charity.

In the interchange of subscriptions given and benefits received, no account seems to be taken of subscribers of less than a guinea. This again is bad business. The small subscriber, and the fact that "many littles make a mickle,' have been too much ignored in many hospitals until lately; but perhaps the wonderful success of the League of Mercy will show the authorities of provincial hospitals the sums they may collect in shillings and in pence. The giver of less than a guinea to the Salop Infirmary got nothing for his subscription, which, especially if he came to realise that the giver of a guinea got four times as much as he paid for, would not incite the small donor to continue his subscription. Probably no donor of half a crown or half a guinea ever worked out the matter statistically, but at the bottom of his mind would be a consciousness that what he gave was given without equivalent, while if he could have afforded to give more he would have had something for his money. If a ticket hospital is to thrive on a business basis every subscriber must get something for his subscription. Not having the rules of the infirmary before us we cannot say if churches, chapels, shops, and factories, where a collection was made for the funds of the infirmary, received tickets in exchange for their subscriptions. Unless they do, they cannot be expected to subscribe. Once the principle of tickets for subscriptions is introduced it should be carried out fairly and logically.

This principle Sir Henry Burdett laid down in his report, for, although he clearly showed that his ideal of a hospital was a free institution, where no claim but necessity was recognised, and where that was never ignored, he laid down certain lines as those on which a ticket hospital might successfully be worked. These lines were for the most part the ones which are followed in the Birmingham General Hospital—"an institution deservedly occupying the first position on account of the excellence of its management and the financial results secured." There the qualification for a governor is a payment of £25, or an annual subscription of two guineas. Each subscriber receives one in-patient letter or 12 out-patient letters for every two guineas contributed. Following out this proportion, three out-patient letters are given for every half-guinea annual subscription, and as 12 out-patient letters are regarded as equivalent to an in-patient one, and will be received in its place, as entitling the person who presents it to in-patient treatment, the subscriber of half a guinea has the assurance of receiving his money's worth in patronage as much as the larger donor. Sir Henry advised the Salop authorities not merely to follow the example of Birmingham, but to "go one better by granting one out-patient letter to each subscriber of 5s. per annum." These gentlemen, however, have not followed this suggestion, and they have now arranged to give two out-patient tickets for each half-guinea subscribed, and four for every guinea. They have also accepted the interchange of out and in tickets, in the proportion of eight out-patient tickets for one in. A life trustee—that is, a donor of thirty guineas in one sum, will receive annually one in-patient or eight out-patient tickets. Out-patients are to receive advice and medicine for six weeks for one ticket; if longer treatment is needed, a new order will be required. This raising of the price of an in-patient ticket—if one may so term it—will help to relieve the pressure on the funds of the hospital, while the fact that out-patient tickets can be used to contribute to an in-patient one, will prevent this increase from discouraging guinea subscribers, and the extensions of the tickets to half-guinea subscribers should win these in considerable numbers.

Yet these changes cannot be said to solve the problem, though they may to some extent relieve the tension. The Salop Infirmary has been going on the principle of reckoning its legacies as ordinary income, and using them accordingly. There is no source of income so uncertain as legacies, and if these windfalls are excluded, the hospital has been for years past spending rather more than £1,000 in excess of its income. If it could be hoped that all the guinea subscribers would double their subscriptions in order to retain their privileges as recipients of in-patient tickets, the matter might be considered arranged; but though some will do so, it cannot be expected that all will. And it must be remembered that the same number of guinea subscriptions will in future admit eight out-patients instead of four, so that the profit on each will fall from three shillings and sixpence to a shilling. Therefore the ultimate hope of the institution must lie in enlarging the area of subscribers. Nor does this seem impossible. Sir Henry said in his report that in his judgment, "what the Salop Infirmary most needs is a quickening of the intelligence of the inhabitants of the town of Shrewsbury and the county of Salop to the vast benefits conferred upon the inhabitants by this institution, and the paramount necessity, in the interest of all classes, that it shall be maintained in the highest state of efficiency." There are signs that this awakening has begun. At the meeting of the trustees at which Sir Henry's report was read and discussed, General Herbert announced that whereas "the average amount of benefactions for the year was generally between £500 and £600, since July 1st (1902) those benefactions had increased to £1,983." This shows that Salop needed only to be told of the needs of its hospital. The gallant General said indeed that "they could not count on that sort of thing as a permanent income." Perhaps that depends to a large extent on whether or not the trustees of the hospital keep its needs before the eyes of the county. Sir Henry suggested that a series of meetings should be arranged in the most populous portions of the county as well as in the town of Shrewsbury, at which the benefits conferred by its existence might be brought before the minds of all. This would probably lead to the formation of village committees, whose members would collect small subscriptions for the infirmary. Hitherto the hospital authorities seem to have trusted too much to the guinea subscribers. The guineas can as a rule be gathered in without much trouble, but their number is limited. But if voluntary workers can be found who will go out and collect the crowns and half-crowns of those of moderate means, the shillings of the tradespeople and even the pence of the cottagers, there will be not only a substantial addition to its funds, but a quickening of interest in its work which will be wholly beneficial to it, and may finally enable it to reach the true ideal—the free hospital.

